# Evolutionary analysis of landscape ecological risk in Baili Rhododendron National Forest Park

**DOI:** 10.1371/journal.pone.0317851

**Published:** 2025-01-24

**Authors:** Yin Yu, Wengang Cui, Suihua Liu, Ting Yu, Yang Song

**Affiliations:** 1 College of Geography and Environmental Science, Guizhou Normal University, Guiyang, China; 2 Guizhou Mountain Resources and Environmental Remote Sensing Application Laboratory, Guiyang, China; Central University of Punjab, INDIA

## Abstract

It is significant to research the ecological risk of land use landscape to promote ecological conservation and restoration. The characteristics of land use dynamic change in Baili Rhododendron National Forest Park were analyzed based on GlobeLand30 data for three periods in 2000, 2010 and 2020. With the support of the landscape ecological risk evaluation model and spatial analysis methods, the features of spatial and temporal differentiation of ecological risk and its spatial correlation in the study area were evaluated. The results show that (1) the landscape pattern changed visibly from 2000 to 2020, the comprehensive land-use dynamics increased from 8.53% to 9.66%, and the land use dynamics are greatest and change most dramatically on construction sites; (2) the landscape ecological risk level of the study area as a whole showed a declining trend, and 96.82% of the ecological risk plot index decreased, and the risk distribution was dominated by lower risk, medium risk and higher risk areas; (3) landscape ecological risk was spatially positively correlated across all time periods, and the spatial aggregation was gradually weakened, with the distribution of LH and HL areas dispersed and the distribution of HH and LL areas concentrated. The overall landscape ecological risk index has witnessed a decline, indicating a positive trajectory in the development of the ecological environment.

## Introduction

Changes in land use serve as a crucial carrier of human impacts on the Earth’s surface, providing a direct depiction of landscape pattern characteristics and reflecting changes in regional ecological environments [1]. Unreasonable land use patterns and development intensity can cause damage to regional ecosystems, especially in ecologically fragile areas [2]. National forest parks encompass rich natural and cultural resources; however, factors such as excessive development and environmental pollution have led to land degradation and sharp declines in biodiversity, posing significant threats to ecological risk and quality. In the current context of accelerating industrial development and urbanization, ecological and environmental problems are becoming more and more prominent as a result of the increasing intensification of contradictions in human-land relations [3], and problems such as landscape fragmentation [4, 5], forest and grassland degradation [6, 7], rocky desertification [8] and environmental pollution [9, 10] are constantly emerging. In the face of such challenges, the construction of ecological civilization must be prioritized. National forest parks, as the primary carriers, can explore the dynamic changes in land use and ecological risks, providing scientific evidence for achieving coordinated development between regional development and ecological environment.

Landscape pattern refers to the spatial distribution and combination of landscape elements of different sizes and shapes [11], and the evolution of landscape pattern can reflect the pattern of change in different periods of the landscape, which has an important impact on the structure and function of regional ecosystems [12]. Landscape ecological risk refers to the possible adverse effects of natural or anthropogenic disturbances on the interaction between landscape patterns and ecological processes [13], and the study of the spatial and temporal evolution of landscape ecological risk is of great significance to improve the quality of regional ecological environment [14]. In 1992 the U.S. Environmental Protection Agency defined ecological risk assessment (ERA) as “the process of assessing the likelihood of ecological impacts from exposure to one or more environmental stressors” (https://www.epa.gov/risk/ecological-risk-assessment) [15]. ERA, as a vital tool for ecological environmental management [16, 17], can provide important scientific information to environmental decision makers [18] With the passage of time, theories and methods of ERA have been enriched, and a more complete evaluation framework has been formed [19], and its development trend is manifested in traditional EAR, regional ERA and landscape ERA [20]. There are primarily two methods for landscape ecological risk assessment. The first method is based on the risk source-receptor-exposure-risk assessment model, focusing on unidirectional ecological receptor studies of different environmental receptors such as water [21], atmosphere [22], and soil [23]. This method emphasizes identifying risk sources and receptors, and the subjective determination of index factors and risk sources during the evaluation process can affect the accuracy of the assessment results. The second method involves ecological risk assessment from the perspective of landscape ecology, utilizing remote sensing (RS) and geographic information system (GIS) technologies. It constructs multi-scale landscape ecological risk assessment models for provinces, cities, and counties based on land use changes and landscape pattern indices [24, 25]. This method considers scale effects, temporal changes, and regional spatial heterogeneity, making it widely applicable and promoting spatial visualization of results. The landscape ERA approach based on land use change has been widely used [26–31], where changes in land use patterns and intensities can respond to spatial changes in the composition and characteristics of landscape ecological risks [32]. In addition, land use change has been shown to be associated with ecological problems such as land degradation, biodiversity loss and ecological vulnerability [33]. Therefore, evaluating the effects of different landscape changes on ecological risks is highly relevant.

Currently, the spatial scale of landscape ecological risk assessment mainly focuses on large-scale regions such as counties [34] and river basins [35], and there is still room for in-depth research on the landscape risk status of ecologically more fragile natural zones, especially in localized areas of national forest parks. Furthermore, previous studies have mostly used administrative divisions as the basic evaluation units, artificially dividing the consistency and integrity of ecosystems, leading to significant errors in assessment results. In turn, when grid cells are used as evaluation units, they are limited by large computational requirements. Each grid cell is required to perform independent ecological risk calculations, which poses bottlenecks in computational resources, complexity in model construction and computation, data processing challenges, and potentially reduced precision and utility of assessment results due to resource constraints. In view of this, this study utilizes ArcGIS Model Builder and Fragstats batch processing tools to construct a cross-platform, process-based large-scale landscape ecological risk assessment model, which can be used as a reference for large research areas experiencing exponential increases in workload.

**Fig 1:**
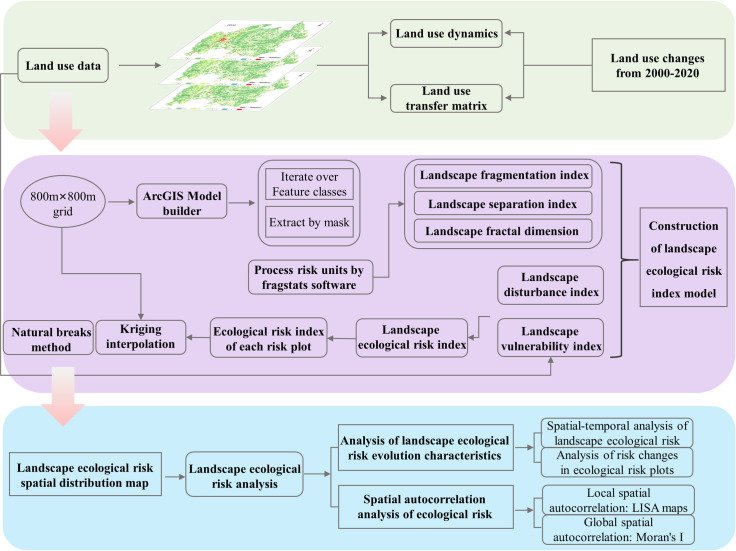
The framework of this study.

Baili Rhododendron is the only national forest park and nature reserve in China to protect Rhododendron spp. of the Rhododendron family [36], and it is a rare forest ecosystem and gene pool of rhododendron germplasm resources in the world with special conservation value, which is of high scientific research value and tourism value [37]. With the development and utilization of forest parks, the contradiction between the limited nature of ecological resources and human needs has become increasingly strong [38], and rational planning and protection of land resources to promote ecological sustainable development has become a key issue [39]. By analyzing the dynamic changes of the landscape pattern of Baili Rhododendron and the ecological risk effect of landscape pattern evolution, it helps to prevent and resolve ecological environmental risks, and is of great significance to optimize the land use layout, improve the regional ecological environment and landscape ecological construction. Based on the above analysis, this study establishes a landscape ecological risk calculation tool by decomposing the landscape ecological risk evaluation process based on the landscape pattern index and applying the ModelBuilder tool of ArcGIS to realize the process, rapidity, and batching of the landscape ecological risk evaluation; and takes the 2000, 2010, and 2020 global surface coverage products GlobeLand30 in 2000, 2010 and 2020 as the main data source, based on the characteristic landscape pattern and its changes in Baili Rhododendron, we comprehensively use remote sensing and GIS technology to analyze the spatiotemporal dynamic changes in landscape ecological risk over the past 20 years in the study area, with the goal of providing scientific basis for the management and coordinated development of the Baili Rhododendron National Forest Park. The basic framework of the study is shown in Fig 1.

## Materials and methods

### Study area

**Fig 2:**
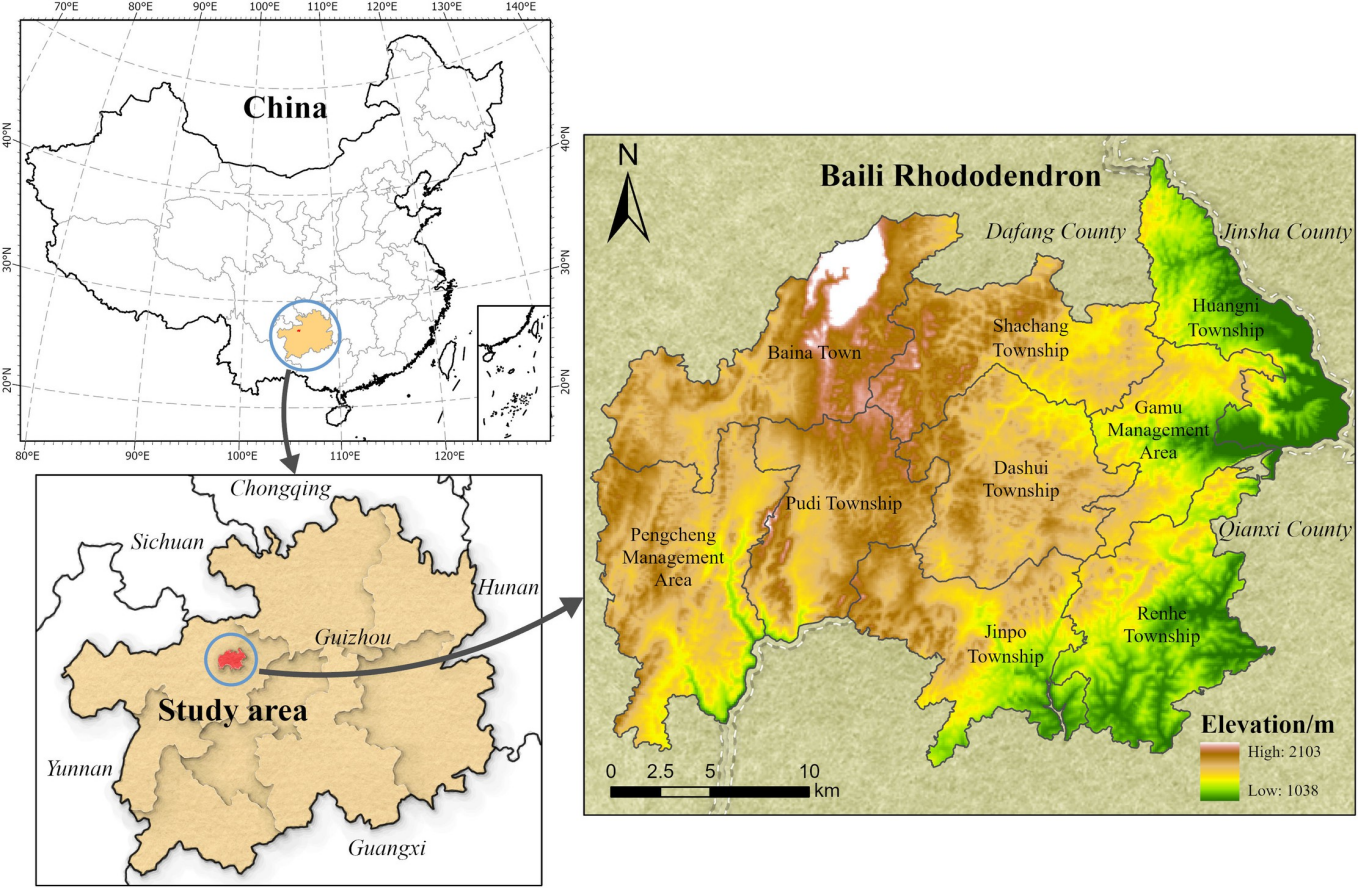
Overview of the study area. This figure was created with ArcGIS Pro (https://pro.arcgis.com/).

Baili Rhododendron National Forest Park (27°10′-27°17′N, 105°50′-106°04′E) (Fig 2) is located in the hinterland of the Wumeng Mountains in the central part of the Bijie Experimental Zone in the northwest of Guizhou Province, China. It boasts the largest area, the most diverse species, and the most intact original rhododendron forest globally. The park is rich in mineral resources, with significant reserves of coal, sulfur, and iron. The predicted coal reserves exceed 1 billion tons, with proven reserves reaching 530 million tons. Situated in the watershed area of the Yachi River, Liuchong River, and Chishui River, it features typical karst landforms, predominantly characterized by high plateau hills and mountains. The climate is classified as a subtropical warm and humid climate, with an annual precipitation of 1150.4mm and an average relative humidity of 84%. The park’s rivers are characterized by mountainous rainwater sources, with short flow processes, narrow watersheds, poor water retention capabilities, and weak regulation functions. Baili Rhododendron National Forest Park is designated as a National Ecotourism Demonstration Zone and a National 5A Tourist Attraction. It serves as a prime example of poverty alleviation through tourism development in Bijie and even Guizhou Province. In recent years, human activities have led to significant changes in the park’s landscape pattern. Conducting landscape ecological risk assessments through land use changes is of great significance for maintaining the ecological health of the Baili Rhododendron National Forest Park and optimizing its ecological risk management.

### Data sources

The primary data source for this study is the GlobeLand30 global land cover product (http://www.globallandcover.com/), with a spatial resolution of 30 meters. This dataset has an overall accuracy of 82.39% and was developed by China and provided to the United Nations [40]. GlobeLand30 data has been widely used in studies of land cover change [41]. Following the classification standard of "Classification of Land Use Status" (GB/T21010-2007) at Level 1, the data is categorized into six types: forest land, arable land, grassland, shrubland, water bodies, and built-up land. DEM (Digital Elevation Model) data was obtained from the Geographic Spatial Data Cloud (http://www.gscloud.cn/).

### Risk subdivision

**Fig 3:**
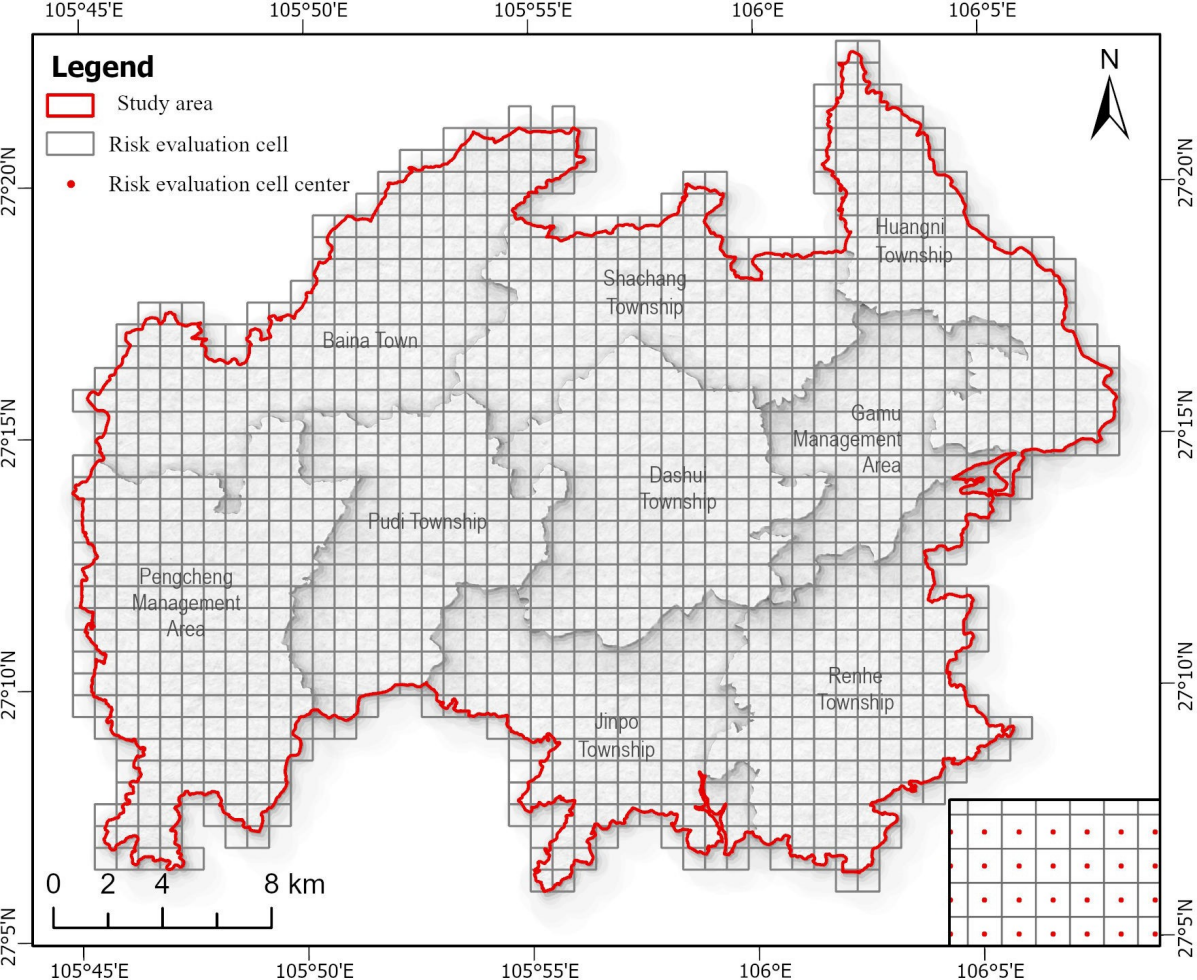
Sketch Map of ecological risk plot division in the study area. This figure was created with ArcGIS Pro (https://pro.arcgis.com/).

To visually represent the landscape ecological risk index, the study area is divided into grids using the equal-interval systematic sampling method, with ecological risk plots as the basic evaluation units. To comprehensively reflect the landscape spatial pattern around the sampling points, the area of ecological risk plots should be 2 to 5 times the average area of landscape patches. The study area is located in the mountainous southwest region, characterized by highly fragmented landscapes such as croplands and forests. Therefore, considering computation intensity, accuracy, and the actual situation of the study area, the study area is divided into 1225 risk plots using 800m × 800m grid cells (Fig 3). The geometric center of each grid cell is designated as a sampling point. The landscape ecological risk index is calculated for each sample area and assigned to the sampling points for spatial interpolation analysis.

### Land use dynamic degree

The dynamic degree of land use is an indicator that measures the difference in the rate of land use change in a region. It can be divided into two types: the dynamic degree of single land use type and the dynamic degree of comprehensive land use. The specific models for both are distributed as follows [42]:

$$L=\frac{U_{b}-U_{a}}{U_{a}}\times \frac{1}{T}\times 100\%$$ (1)

where *L* is the dynamic degree of a certain land use type in the research period; *U*_a_ and *U*_b_ are the area (km^2^) of a specific land use type at the beginning and end of the study, respectively; *T* is the research time (years).

$$LUD=\left\{\sum_{ij}^{n}\left(\frac{\Delta U_{i-j}}{U_{i}}\right)\right\}\times \left(\frac{1}{t}\right)\times 100\%$$ (2)

where *LUD* is the dynamic degree of comprehensive land use; *U*_*i*_ is the area of the i-type land use at the initial time; Δ*U*_*i-j*_ is the total area (km^2^) of the i-type land use that is converted to other land use types in the t period.

### Landscape ecological risk index model

Landscape ecological risk assessment involves analyzing the degree to which human activities, natural disasters, and other disturbance factors affect the landscape from the perspectives of landscape structure, pattern evolution, or landscape ecological processes [43, 44]. In the context of the Baili Rhododendron National Forest Park, this study constructs a landscape ecological risk index model based on the degree of landscape ecological loss. By referring to relevant literature and analyzing related landscape indices, the risk values for each evaluation unit are calculated. The main indicator formulas are as follows:

#### (1) Landscape disturbance index


$$E_{i}=aC_{i}+bN_{i}+cF_{i}$$ (3)

The index of interference degree reflects the extent of damage caused by external disturbances to a particular landscape. *C*_*i*_, *N*_*i*_ and *F*_*i*_ represent landscape fragmentation, landscape isolation, and landscape fractal dimension, respectively. a, b and c are the corresponding weights, and a + b + c = 1. Based on previous research results, the weights are set as a = 0.5, b = 0.3, c = 0.2 [45].

#### (2) Landscape vulnerability index

The ability of different landscapes to maintain biodiversity, protect species, preserve structural and functional integrity, and promote natural succession varies depending on the represented ecosystems. Their resilience to external disturbances also differs [46]. This difference mainly depends on the stage of ecological succession that the ecosystem is in. According to the characteristics of each landscape’s natural succession stage, combined with relevant research results, the six types of landscapes are ranked from high to low as follows: water area = 6, cultivated land = 5, grassland = 4, shrub land = 3, forest land = 2, construction land = 1, and then, they were normalized to obtain each vulnerability degree(*F*_*i*_) [47].

#### (3) Landscape Ecological risk index

To construct the Landscape Ecological Risk Index (ERI) model, the aforementioned landscape indices are selected. This model aims to describe the comprehensive magnitude of ecological losses within each assessment unit, with the intention of transforming landscape spatial patterns into spatialized ecological risk variables through sampling [48]. The calculation formula for the Landscape Ecological Risk Index (ERI) is as follows:

$$ERI_{k}=\sum_{i=1}^{n}\frac{A_{ki}}{A_{k}}\sqrt{E_{i}\times F_{i}}$$ (4)

Where, *A*_*ki*_ is the area of landscape i in sample region k, *A*_*k*_ is the total area of sample region k, $\sqrt{E_{i}\times F_{i}}$ is loss index of landscape, n is the number of landscape type.

### Spatial autocorrelation analysis

The Landscape Ecological Risk Index is a representative quantitative indicator exhibiting spatial heterogeneity patterns. Spatial autocorrelation analysis is employed to discern the inherent correlations of a variable in space [49], and it can be measured using two types of indicators: global and local Moran’s I. In this study, Geoda 5.3 was utilized for spatial autocorrelation analysis of the Landscape Ecological Risk Index.

The global Moran’s I statistic can determine the spatial patterns of ecological risk in the Baili Rhododendron landscape as a whole. The value greater than 0 indicates positive correlation and tendency towards clustering; less than 0 indicates negative correlation and dispersion; while the value equal to 0 suggests no correlation and random distribution [50]. Local Moran’s I is employed to reflect the degree of correlation between the attribute values of small local units and those of neighboring areas. The results can generate LISA cluster maps, which reflect the spatial correlation and distribution of landscape ecological risk [51].

## Results

### Analysis of land use change

#### Temporal and spatial patterns evolution of land use

**Fig 4:**
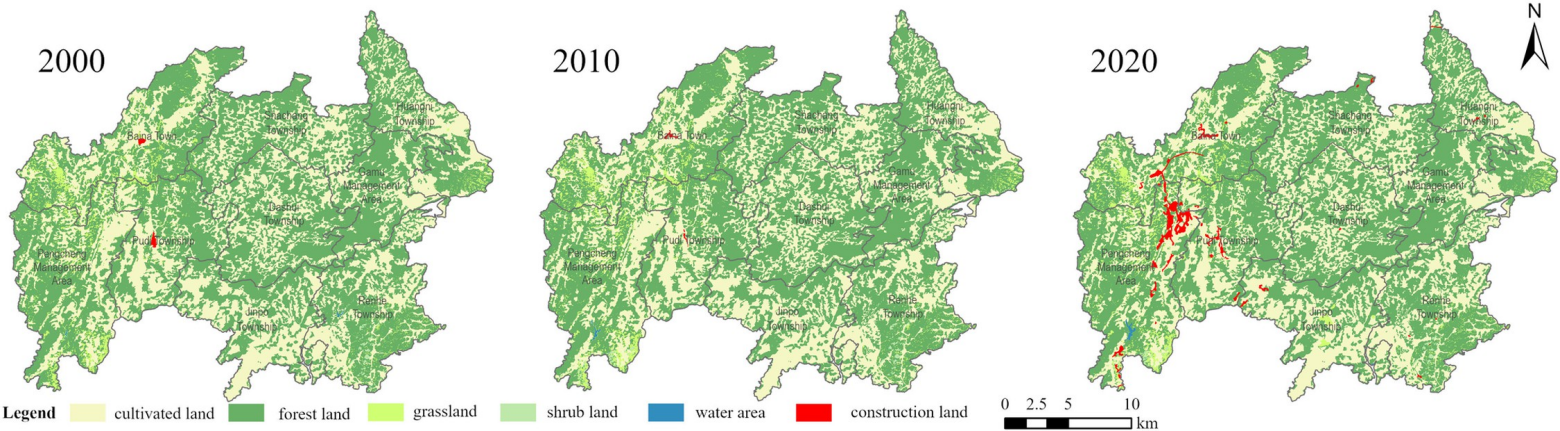
Landscape patterns of land use types in 2000, 2010 and 2020. These maps were created by reclassification based on GlobeLand30 data and showed land use types of the region in 2000, 2010 and 2020. These figures were created with ArcGIS Pro (https://pro.arcgis.com/).

**Fig 5:**
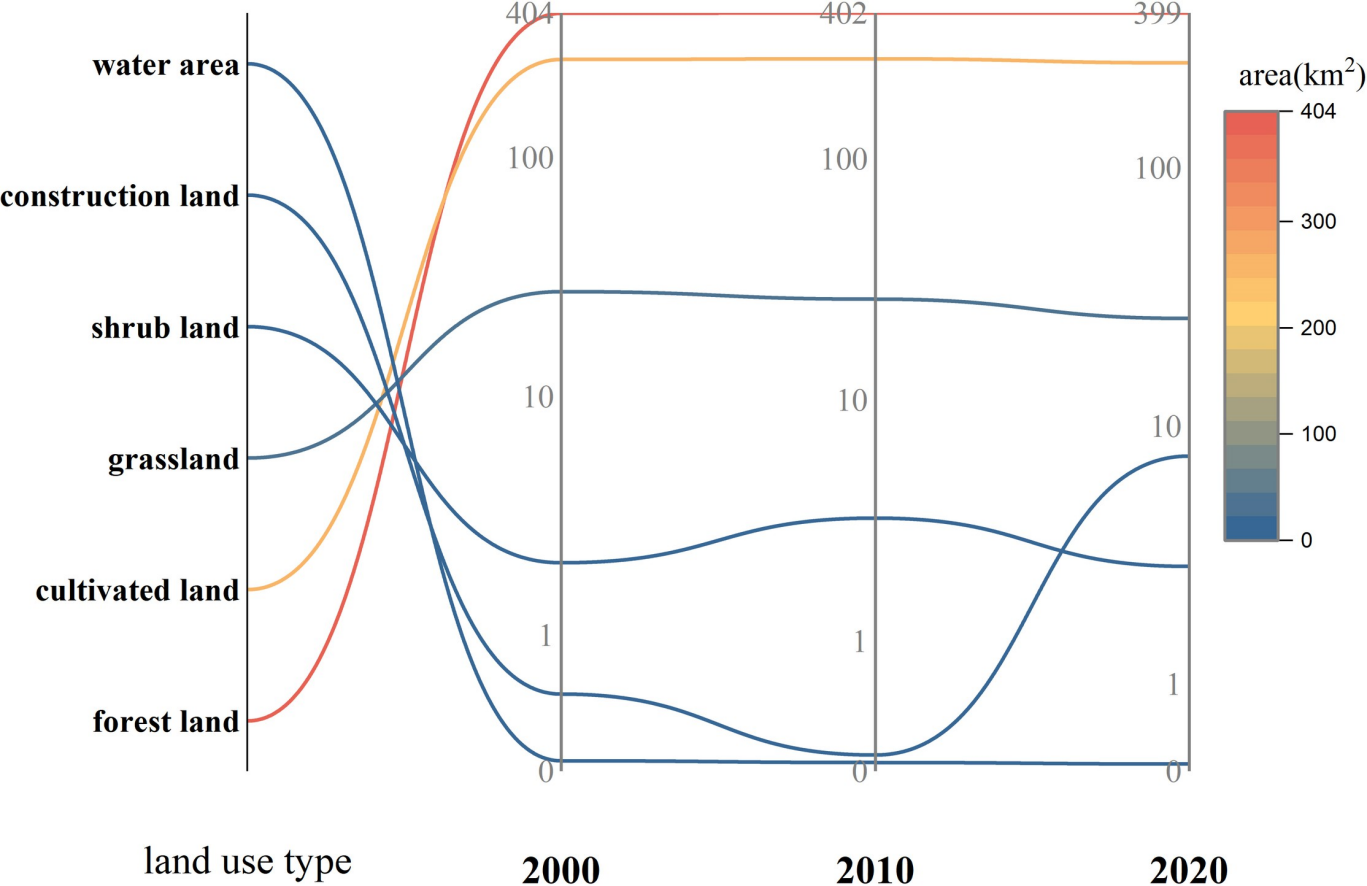
Area of land use types from 2000 to 2020.

The overall distribution and changes of various land types in the Baili Rhododendron National Forest Park in 2000, 2010, and 2020 are illustrated in Figs 4 and 5. From 2000 to 2020, there were slight fluctuations in the area of different land use types within the park. Specifically, forest, cultivated land, and grassland areas decreased slightly, while water, construction land, and shrub land areas all showed slight increases. Notably, there was a significant expansion in construction land, particularly centered around the Pengcheng Management Zone, which increased by 7.10 km^2^ by 2020. Forest and cultivated land are the dominant land use types in the Baili Rhododendron area, accounting for approximately 95% of the total area. In particular, in the Baina Town, the cultivated land and forest areas are 32.3 km^2^ and 61.3 km^2^, respectively, while in the Pengcheng Management Zone, they are 39 km^2^ and 60.3 km^2^, respectively. Grassland is mainly distributed in the eastern part of the park (Fig 5). Overall, from 2000 to 2020, the spatial distribution pattern of cultivated land and forest land in Baili Rhododendron remained relatively stable. The forest coverage rate in the entire management area reached 69%, resulting in varying degrees of forest distribution throughout the area. Additionally, the fragmentation degree of cultivated land is correlated with the terrain, with cultivated land being more continuous at higher elevations and more fragmented at lower elevations.

#### Analysis of land use dynamics change

**Fig 6:**
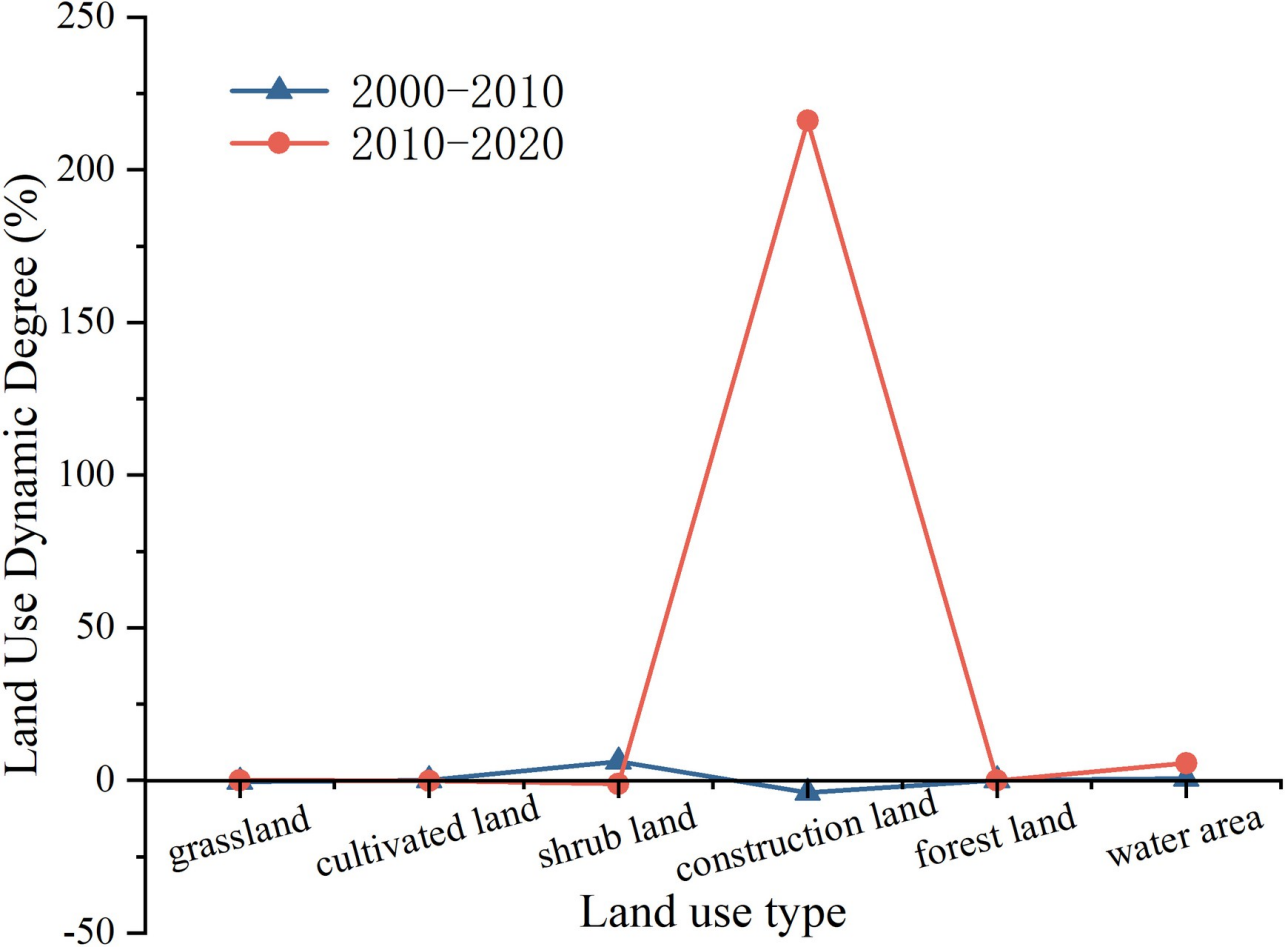
Dynamic degree of single land use from 2000 to 2020.

From the perspective of comprehensive land use dynamics, the comprehensive land use dynamic index in the study area increased from 8.53% to 9.66% between 2000–2010 and 2010–2020 (Table 1). As depicted in Fig 6, during the study period, there were significant differences in the single dynamic index of each land use type. Land use types with notable changes in their single dynamic degree index include construction land, shrub land, and water. From 2000–2010, shrub land and construction land experienced the greatest changes in single dynamic degree index, with shrub land increasing by 6.09% and construction land decreasing by 4.04%. From 2010–2020, except for construction land and water, the single dynamic index of other land use types showed a decreasing trend. Construction land experienced the most significant change in single dynamic index, transitioning from negative to positive, increasing by 216.07%. Grassland and forest land continued to decrease, with the rate of change in grassland slowing significantly compared to the period from 2000–2010, while the rate of change in forest land accelerated compared to the previous decade. Shrub land and cultivated land shifted from initially positive changes to negative changes, while water area continued to grow positively. Overall, the area and distribution of all land use types in the study area have changed over the past 20 years, with construction land undergoing the most significant changes. This is because since being designated as a regional-level nature reserve in 2001, the Baili Rhododendron National Forest Park has gradually improved supporting facilities. The opening of the China-Guizhou International Baili Rhododendron Flower Festival in the Baili Rhododendron National Forest Park in 2009 prompted changes in the landscape pattern. Human interference intensified, leading to an acceleration in the rate of change in land use types, with construction land changing more rapidly than other land use types.

**Table 1:** Comprehensive land-use dynamics, 2000–2010 and 2010–2020.

Time period	2000–2010	2010–2020
Comprehensive land-use dynamics(%)	8.53	9.66

#### Analysis of land use transfer change

**Fig 7:**
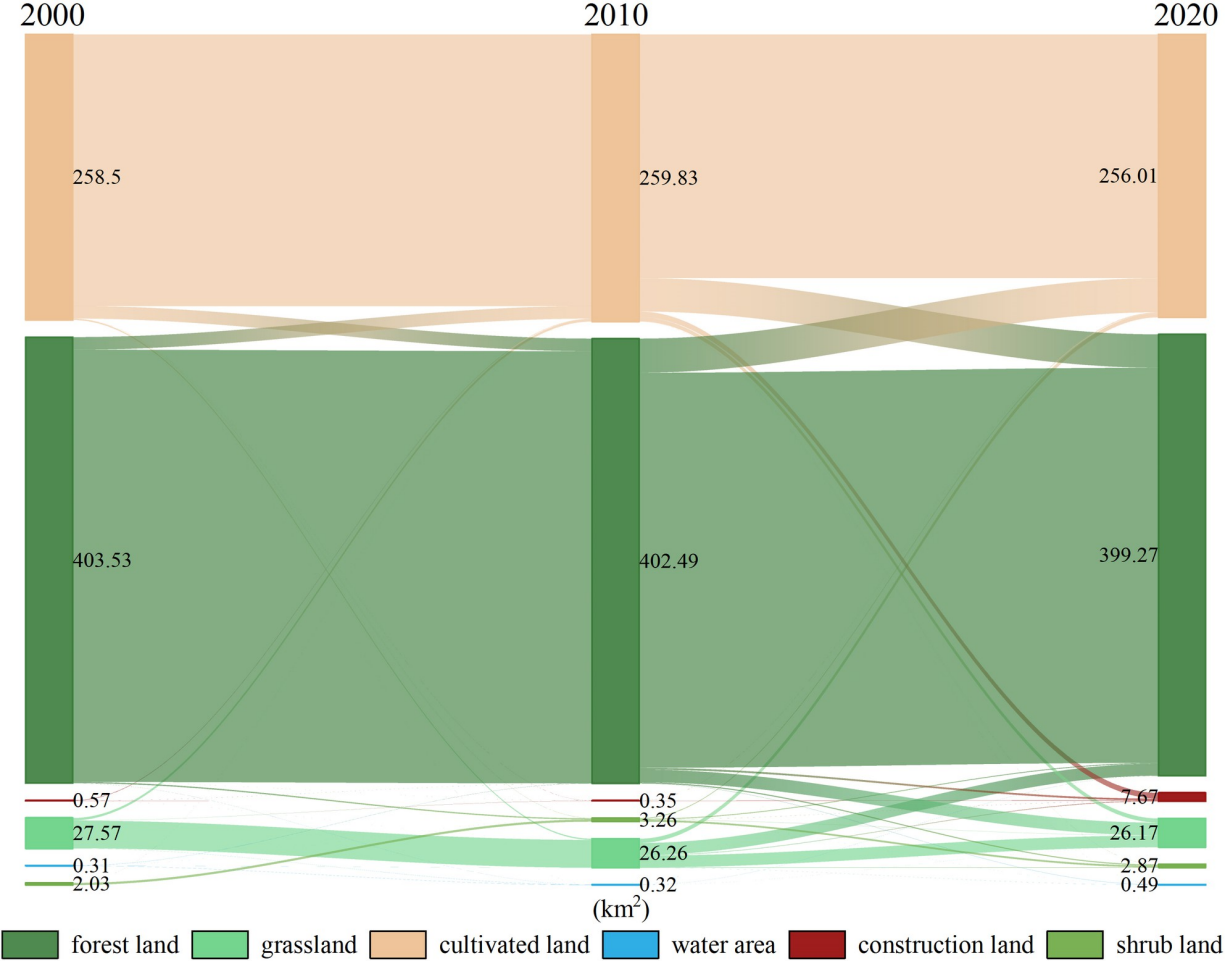
Land use landscape transfer area matrix from 2000 to 2020.

To further visualize the spatial evolution characteristics and mutual transformation rules of different land use types in the Baili Rhododendron area, GIS spatial analysis techniques and the land use transfer matrix model were employed (Fig 7). This analysis aimed to analyze the direction and quantity of changes between different land use types. From 2000 to 2020, forest land was the main land use type that converted to other types. Forest land mainly transformed into cultivated land and grassland, with conversion areas of 29.14 km^2^ and 11.64 km^2^, accounting for 65.92% and 26.32% of the total conversion area, respectively. Forest land also constituted the main land use type formed from conversion, predominantly transforming into grassland and arable land, with conversion areas of 11.33 km2 and 27.87 km2, accounting for 28.36% and 69.73% of the total conversion area, respectively. Additionally, as one of the top ten natural ecotourism attractions in Asia and the Greater China region, the study area possesses a favorable natural background and ecological resources. However, the development and construction activities have led to the erosion of ecological land, posing certain ecological risks to the study area.

### Analysis of landscape ecological risk evolution characteristics

#### Spatial-temporal analysis of landscape ecological risk

**Fig 8:**
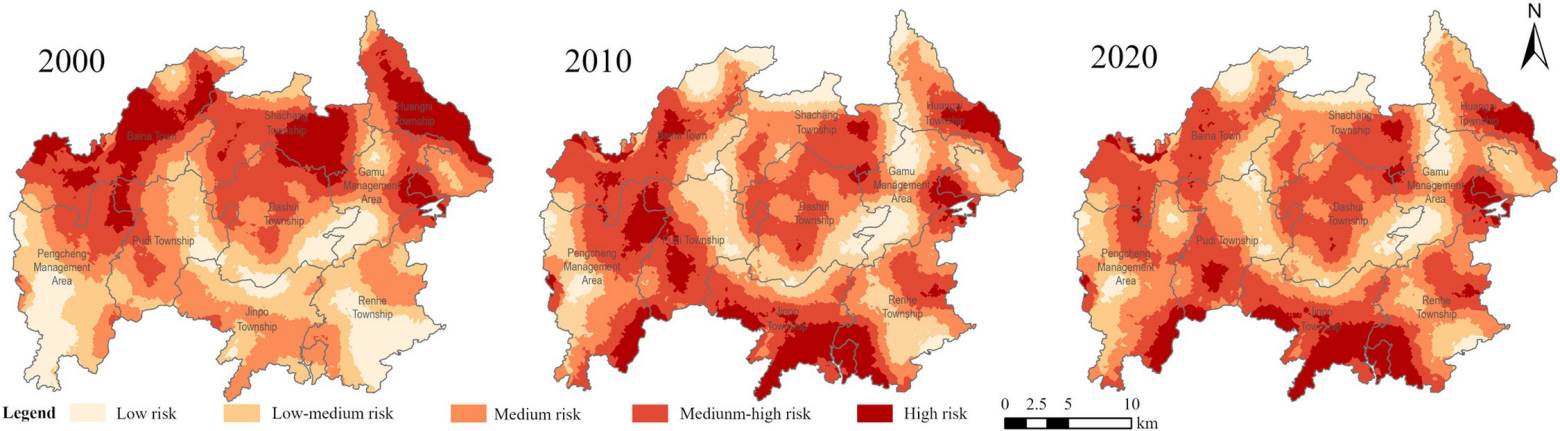
Landscape ecological risk classification of Baili Rhododendron National Forest Park in 2000–2020. These maps were created with ArcGIS Pro (https://pro.arcgis.com/).

The spatial interpolation of landscape ecological risk indices of Baili Rhododendron National Forest Park across three stages was conducted, and the Jenks natural classification was utilized to classify them into five levels. The spatial distribution maps of landscape ecological risk from 2000 to 2020 were obtained (Fig 8), and the area and proportion of each risk level were calculated (Table 2).

**Table 2:** Ecological risk level and area from 2000 to 2020.

Ecological risk level	2000		2010		2020	
	area/km^2^	percentage/%	area/km^2^	percentage/%	area/km^2^	percentage/%
Low risk	85.62	12.36	52.76	7.61	49.55	7.15
Low-medium risk	155.92	22.50	146.80	21.18	143.55	20.71
Medium risk	190.27	27.46	182.49	26.33	203.73	29.40
Medium-high risk	162.90	23.51	221.20	31.92	224.28	32.36
High risk	98.27	14.18	89.73	12.95	71.88	10.37

From a spatial distribution perspective (Fig 8), the high-risk areas were observed to exhibit a trend of shifting from north to south. Subsequently, the low-risk areas were mainly distributed in the surrounding areas of Shachang Township, Baina Town, and the Gamu Management Zone. These townships were predominantly characterized by grassland and forest land, with a higher vegetation coverage and a lower degree of landscape fragmentation. Consequently, the level of landscape ecological risk remained relatively stable. The medium-risk areas were primarily concentrated in regions with steep slopes, characterized by complex terrain and significant fluctuations in topography, resulting in a higher vulnerability of the landscape. On the other hand, the medium-high risk areas were predominantly distributed in most parts of Baina, Pudi, Jinpo, and Dashui Townships, where various scenic spots and protection units of the Baili Rhododendron Nature Reserve were located. Therefore, these areas were more likely to be affected by human activities, leading to a higher degree of landscape fragmentation. From the trend of landscape ecological risk changes between 2000 and 2020, the overall landscape ecological risk in the Baili Rhododendron National Forest Park was at a medium level, showing a decreasing trend over time. Since the proposal of building a beautiful China with ecological civilization at the 18th National Congress of the Communist Party of China, the Baili Rhododendron management area has actively responded by implementing a series of significant resource protection and restoration projects directly impacting the ecosystem of the Baili Rhododendron National Forest Park. In 2018, the "Regulations on Baili Rhododendron Scenic Area in Bijie City" were formulated to strengthen protection and management, promoting harmonious coexistence between humans and nature. Consequently, the trend of landscape ecological risk demonstrated a benign development. Therefore, it is necessary to coordinate the relationship between economic development and environmental protection in the future.

The proportion of different ecological risk zones in the Baili Rhododendron area from 2000 to 2020 and the temporal variation characteristics of regional ecological risk levels over the past 20 years are shown in Table 2. The data illustrate the area distribution of each ecological risk level. The area of low-risk zones within the study area has consistently been the smallest, with risk distribution primarily comprising low-medium, medium, and medium-high risk zones, totaling over 73% of the total area. Among them, the medium-risk zones are the most predominant type, accounting for over 26% of the total area, followed by the medium-high risk zones, with a proportion ranging from 23% to 33%. The high-risk zones occupy more than 10% of the total area, while the proportion of low-risk zones has decreased to less than 10%. Over the 20-year period, significant changes have occurred in the ecological risk zones, with the areas of medium and medium-high risk zones showing an upward trend. Specifically, the area of medium-risk zones decreased from 190.27 km^2^ in 2000 to 182.49 km^2^ in 2010, then increased to 203.73 km^2^ in 2020. The area of medium-high risk zones continued to rise before stabilizing, while the area of low- medium risk zones continued to decrease before stabilizing. Throughout the entire study period, the areas of low-risk and high-risk zones continuously decreased, with the area of high-risk zones decreasing by 26.39 km^2^.

#### Analysis of risk changes in ecological risk plots

**Fig 9:**
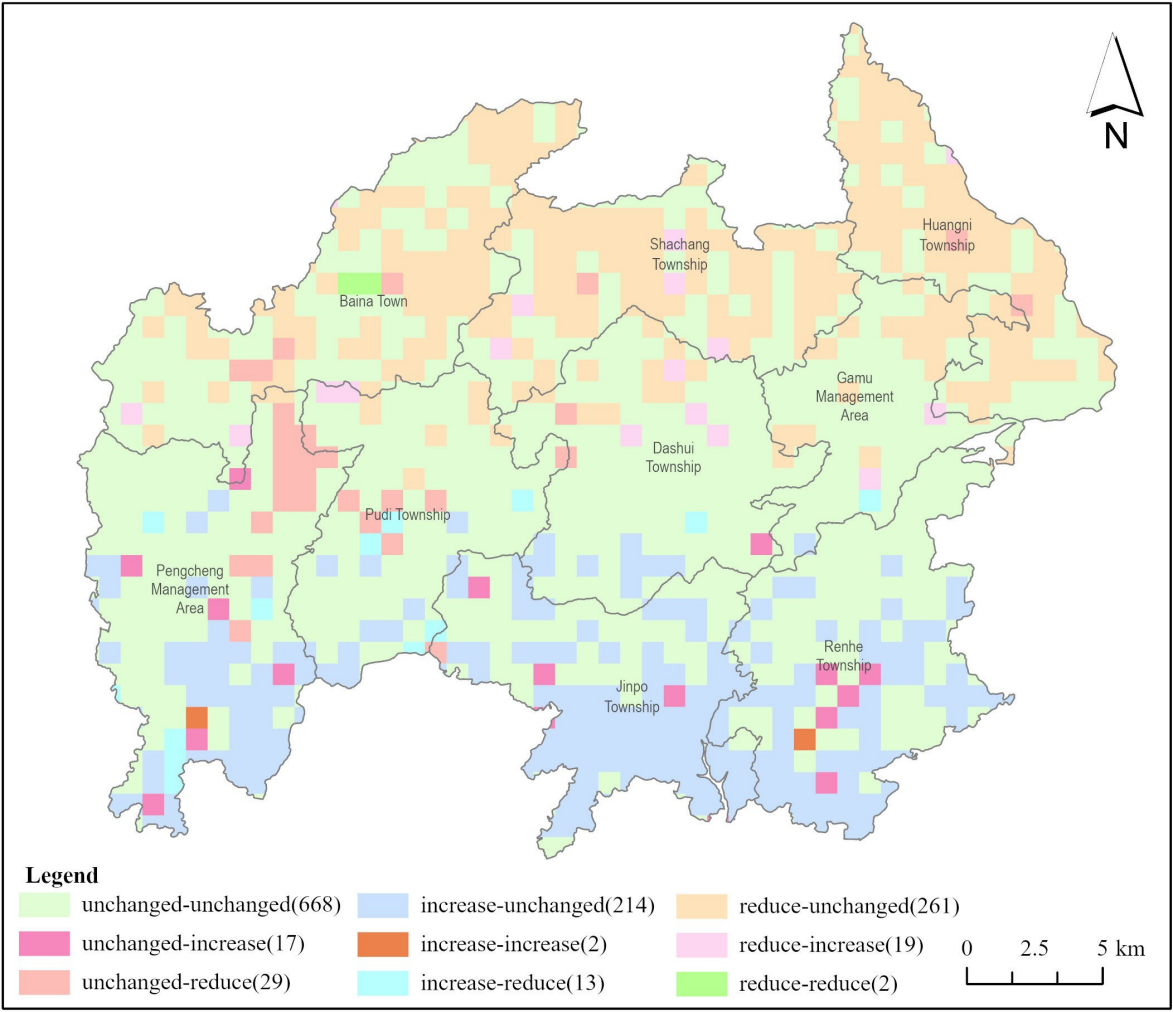
Changes in risk values of each ecological risk plot from 2000–2010 and 2010–2020. The maps were created with ArcGIS Pro (https://pro.arcgis.com/).

Utilizing Eqs (3) and (4) and with the assistance of Fragstats software, the landscape ecological risk index (ERI_k_) for the 1225 ecological risk plots in the study area was calculated for three time periods. The average risk values in the study area from largest to smallest across the three time periods were as follows: 2000 (0.14~0.59) > 2010 (0.12~0.48) = 2020 (0.12~0.48), with an approximate average of 0.20, indicating insignificant changes in risk values. From 2000 to 2010 and from 2010 to 2020, the majority of risk subzones (668) maintained unchanged risk values, accounting for 54.53% of all ecological risk subzones. Following this, there were 261 risk subzones where the risk values decreased first and then remained unchanged, accounting for 21.31%. 214 risk subzones showed an initial increase followed by stability, constituting 17.47% of all risk subzones. Among them, 2 risk subzones demonstrated continuous increases in risk indices, while 2 risk subzones showed continuous decreases in risk indices (Fig 9). Overall, from 2000 to 2020, the risk indices decreased in 1186 risk subzones while increased in 33 risk subzones. From a spatial distribution perspective, the ecological risk subzones with unchanged risk values were primarily located in the central region of the study area, including Pudi Township, Dashui Township, and the Gamu Management Zone, indicating stable internal landscape structures and strong resistance to disturbance. In the southern part of Baili Rhododendron, including Shachang Township, Huangni Township, and Baina Town, most of the risk values exhibited a decreasing trend. This area, characterized by higher altitudes, experiences relatively weaker human-induced landscape modifications and less anthropogenic disturbances. The ecological risk subzones with increased risk values were mainly distributed in Jinpo Township, Renhe Township, and the Pengcheng Management Zone. These areas exhibited relatively higher degrees of landscape fragmentation, weaker stability of ecosystems, and lower resistance to disturbances. Consequently, under the influence of both human activities and natural disturbances, the ecological risk changed rapidly and significantly in these areas.

### Trend analysis of landscape ecological risk evolution

#### Global spatial autocorrelation analysis

**Fig 10:**
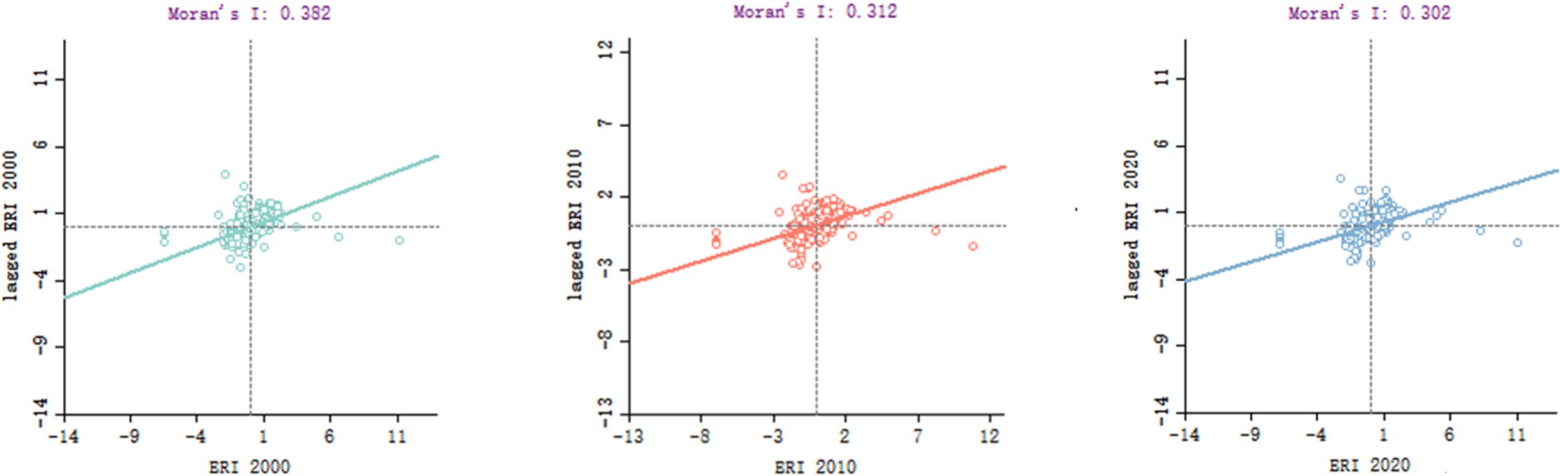
Moran’s I value scatter distribution of the landscape ecological risk index for the Baili Rhododendron from 2000 to 2020.

Moran’s I index was employed to describe the global spatial autocorrelation of landscape ecological risk indices in the study area, as shown in Fig 10. The Moran’s I values were relatively low, ranging from the largest to the smallest for 2000 (0.382), 2010 (0.312) 2020 (0.302), with Z-values of 18.06, 15.13, and 14.45 and P-values were all 0.01, indicating a significant spatial positive correlation in Baili Rhododendron’s landscape ecological risk indices over the 20-year period. Additionally, the distribution of points in Fig 9 closely aligns with the regression line, indicating a clustering trend in the spatial distribution of risk values in Baili Rhododendron. However, Moran’s I values exhibit a decreasing trend from 2000 to 2020, suggesting a gradual weakening of spatial clustering in landscape ecological risk over time.

#### Local spatial autocorrelation analysis

**Fig 11:**
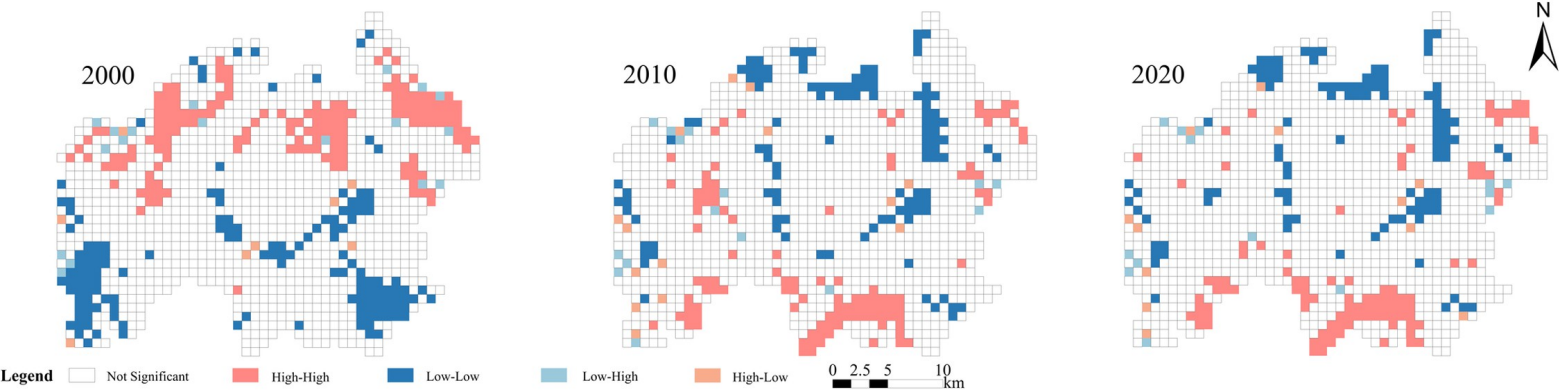
The LISA maps of local spatial auto-correlation in 2000, 2010 and 2020.

The LISA cluster map utilizing the landscape ecological risk index depicts local spatial autocorrelation (Fig 11). In the study area, four significant spatial autocorrelation patterns were observed: high-high (HH), low-low (LL), low-high (LH), and high-low (HL). The HH zones were primarily concentrated in the northern region of Baili Rhododendron in 2000, gradually shifting towards the southern areas over time. LH zones were dispersed around HH areas, predominantly comprising agricultural land. From 2000 to 2020, LH zones gradually dispersed from the northern part of the study area to the surrounding areas due to pronounced human disturbances, resulting in noticeable changes in agricultural land and consequently higher ERI values. LL zones were initially concentrated in Renhe Township, Dashui Township, and Pengcheng Management Area in 2000, forming a ring-shaped distribution in the central part of the study area by 2010 and 2020. HL zones were scattered around LL zones, exhibiting a similar relationship to that between HH and LH zones. However, the key distinction lies in LL and HL zones being predominantly forested land use and land cover (LULC) types, resulting in relatively lower ERI values within forested areas.

## Discussion

### Ecological risk center of gravity transfer

**Fig 12:**
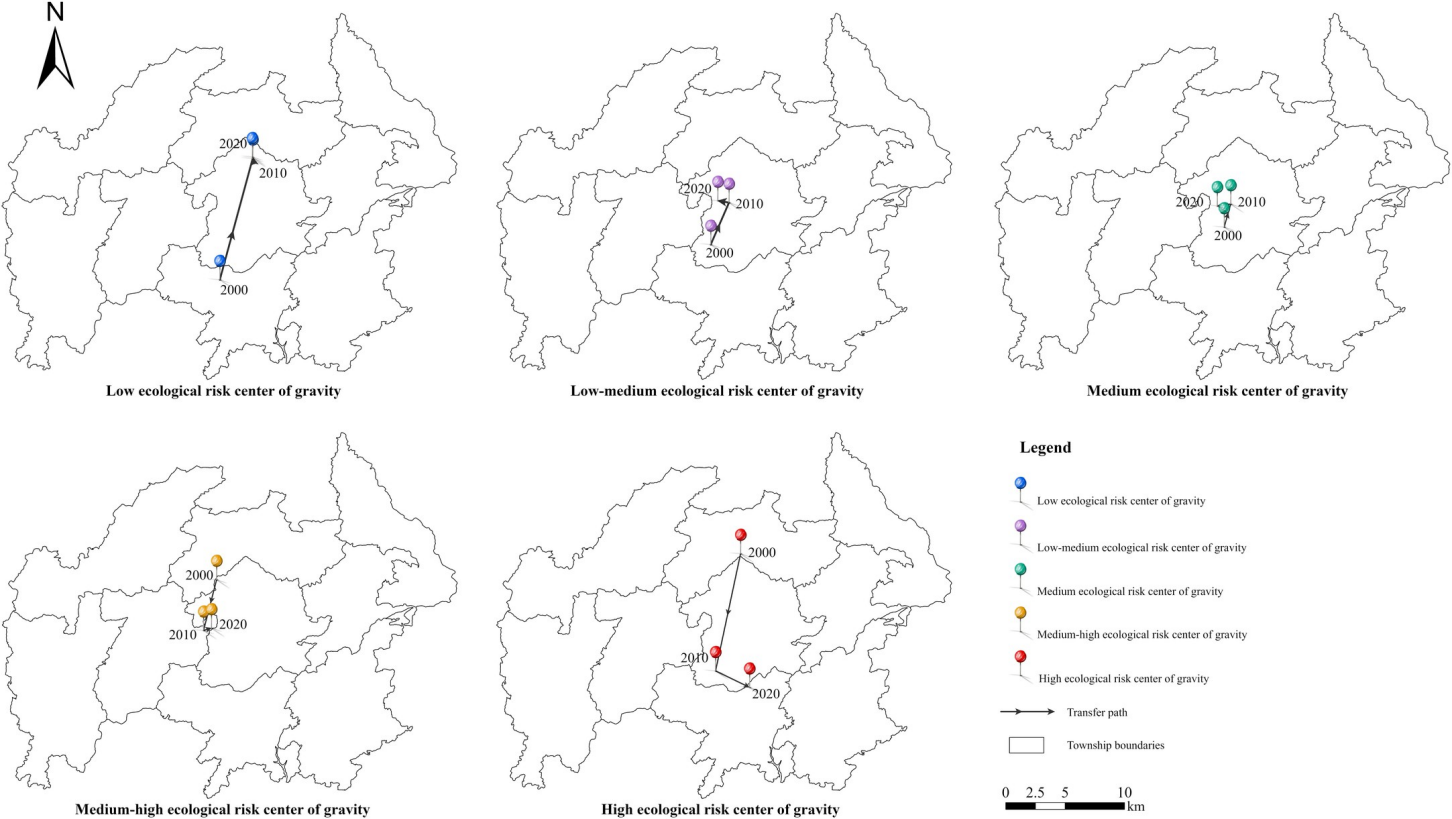
Ecological risk center of gravity transfer in Baili Rhododendron from 2000 to 2020. The maps were created with ArcGIS Pro (https://pro.arcgis.com/).

Based on the characterization of the spatial and temporal evolution of the ecological risk of the Baili Rhododendron, the spatial analysis tool of ArcGIS was used to quantitatively analyze its center of gravity coordinate shift (Fig 12).The research shows that from 2000 to 2020, the overall transfer of the center of gravity of the low-medium, medium-high and medium ecological risk zones changed relatively little, and was mainly concentrated in Dashui Township in the central part of the Baili Rhododendron National Forest Park. Among them, the center of gravity of the low-medium and medium ecological risk zones shifted in almost the consistency direction, both shifted to the northeast and then moved slowly to the west, with the difference that the center of gravity of the low-medium risk zone shifted significantly faster than that of the medium-risk zone. While the center of gravity of the medium-high ecological risk zone had shifted from the northwestern part of Dashui Township to the northeastern part of Pudi Township, with an overall shift of 5.25 km to the southwest from 2000 to 2020. The center of gravity of the high ecological risk zone shifted over the largest spatial span, from the southern part of Shachang Township to the northeastern part of Jinpo Township, and shifted southward by 13.69 km, with the fastest rate of shift from 2000 to 2010, which was 10.18 km to the south. In contrast, the low ecological risk zone showed a trend of shifting from south to north, spatially shifting from the northwestern part of Jinpo Township to the northern part of Dashui Township, and shifting northward by 10.81 km, of which 10.68 km had shifted northward from 2000 to 2010.

It is observed overall that the center of gravity of the low, low-medium and medium ecological risk zones shifted towards the northern part of the study area, and the medium-high and high ecological risk zones shifted towards the southern part of the study area, showing an opposite direction of shift. This is due to the fact that the tourism industry developed in Baili Rhododendron with rhododendron as the core attractive element is highly seasonal, and this seasonality exerts pressure on Bailey’s Azalea’s environmental carrying capacity. Especially the townships of Pudi and Jinpo, as the main sights concentration areas, were affected by human interference and landscape changes, which threatened the ecological balance of the region, increased the area of high and medium-high ecological risk areas, and expanded to the south-west, so the center of gravity of the high and medium-high ecological risk ecological risk areas moved to the south, which led to the center of gravity of the low, low-medium, and medium ecological risk areas to move to the north.

### Analysis of land use change and landscape ecological risk

The relationship between regional ecological risks has been a hot research topic in academia, and land use can directly reflect landscape conditions [52]. Combining the land use change and landscape ecological risk evaluation model, it was found that the landscape ecological risk index of BailiRhododendron National Forest Park showed a decreasing trend, but this is not contradictory to the traditional impression that development and construction lead to ecological deterioration. The ecological risk of the landscape is expressed according to the type and structure of the landscape, which is a problem facing the regional landscape in the future [53]. In the study area, there are a large number of mining areas, which were extremely susceptible to external disturbances, which led to the destruction of surface vegetation near mining areas and induced soil erosion as well as rocky desertification [54], with high vulnerability and high exposure to high risk indices. However, some studies have shown that although built-up land landscapes have low landscape ecological risk, due to the high level of human activities around built-up land, an increase in the area of built-up landscapes increases the landscape ecological risk in the surrounding area [55]. In contrast, forest lands have higher biodiversity. Therefore, under the same disturbance of external factors, forest lands are exposed to higher risks than built-up lands and suffer from a higher degree of landscape vulnerability.

From 2000 to 2020, the land use of Baili Rhododendron National Forest Park had changed, and the land use change was generally affected by both human activities and natural factors, which is consistent with the results of other studies [56, 57]. And human activities could have a significant impact on landscape patterns, previous studies on nature reserves found that human invasion and land exploitation apparently changed the structure and function of the area [58–60]. Land development affects vegetation cover and reduces the continuity and integrity of natural geographic units, leading to a decline in habitat quality in nature reserves [61], which in turn leads to an increase in ecosystem function and landscape ecological risk [62]. In the three study nodes, significant changes occurred among the different risk classes in 2010. The landscape ecological risk index in the study area showed a high level in 2000 and a decreasing trend in 2010 and 2020. Since 2000, the increasingly improved land use system and ecological protection policies have effectively reduced the ecological risks associated with economic growth. At the same time, China has entered a deepening stage of ecological protection, and the National Forest Protection Plan has been vigorously implemented to strictly manage and protect forest nature reserves [63]. A series of ecological construction and protection projects, such as natural forest resources protection, returning farmland to forests, and key protection units, have been launched, effectively curbing ecological environment destruction [64]. The establishment of a long-term mechanism for ecological environmental protection further promoted the coordinated development of the regional economy and ecological environment, the landscape pattern of Baili Rhododendron was continuously optimized, and the overall ecological risk value of the study area was reduced. With the continuous improvement of the land policy system, the pertinence of land planning has gradually enhanced [65]. As a result, the spatial correlation between landscape pattern and ecological risk also decreased year by year.

This study has investigated the spatial and temporal evolution characteristics of the ecological risk of Baili Rhododendron in the past 20 years, which can reflect the long-term trend of the ecological quality of the protected area to a certain extent. In general, the distribution of risk areas was relatively stable, and it could be judged that the ecological environment construction of Baili Rhododendron was effective. However, due to the typical karst geomorphological features of Baili Rhododendron, and the relatively single tree species in the forest area, the forest ecological stability is poor, coupled with the rapid expansion of tourism resources development in recent years has brought about a large number of anthropogenic activities to interfere with the forest ecosystem of the area, which makes the forest ecosystem of the area suffer from a greater impact [66]. Therefore, in order to further improve the ecological carrying capacity of Baili Rhododendron and construct a good ecological environment, it is necessary to continue to implement strict ecological environmental protection policies, and to promote the construction of ecological civilization is the key to promoting the sustainable development of the protected area.

### Ecological conservation and sustainable development

The relevant departments should scientifically plan the development and use of land resources in the Baili Rhododendron National Forest Park, and in the future, priority should be given to protecting the ecological environment of the Baili Rhododendron as far as possible, and to minimizing the damage caused by human activities. In addition, the development and construction of the park should be based on the carrying capacity of the ecosystem, fully recognize the ecological potential of the park, and ensure that the development of tourism construction is coordinated with the ecological protection of the park. For areas with rich scenic sources and ecological sensitivity, core forest and grass resources protection can be strengthened through the establishment of ecological protection zones [67]. Then, the study area accounts for a relatively large area of cultivated land, and the implementation of returning farmland to forest should be increased to accelerate the transformation of cultivated land resources to forest and grass resources. The fragmentation of the landscape pattern should be reduced under the circumstance of ensuring the daily supply of local residents [68], and the moderate implementation of returning small areas of dispersed cultivated land to forests and grasses will facilitate the enhancement of the landscape ecological pattern of the Baili Rhododendron. The changes in the landscape pattern of Baili Rhododendron are mainly due to the increased population flow and industrial restructuring [69, 70]. Therefore, the transformation and destruction of the original ecological environment should be avoided during the planning and construction of the park, and its unique rhododendron ornamental plant resources should be reasonably developed and utilized, and ecotourism should be carried out in a restrained manner to minimize the ecological risks caused by the landscape structure and spatial pattern [71]. Additionally, in order to maintain and improve the ornamental properties of Baili Rhododendron, and to retain the stability of the community, it is recommended to carry out scientific management, such as forest closure, forest window opening, and logging, in order to improve the understory environment, and to ensure the sustainable renewal of the population [72].

### Limitation

There are a few limitations and improvements that need further in-depth research and discussion. Firstly, this study only considered landscape structure changes caused by human activities as sources of risk, overlooking influences from natural factors such as vegetation patterns, meteorological changes, and geological hazards. Hence, future research should comprehensively evaluate landscape ecological risks from multiple sources. Secondly, it only analyzed the spatiotemporal evolution of existing land use data, without considering potential future changes in regional land use. Therefore, the next step should involve predictive studies. Finally, the issue of modifiable areal unit problem (MAUP) was not considered, which can also affect the analysis results in ecological studies. Future efforts should actively seek methods to address this problem.

## Conclusions

The development and regional economic growth have brought significant changes to the land use in the Baili Rhododendron National Forest Park, leading to complex impacts on the regional landscape structure and pattern. The research findings indicate: (1) From 2000 to 2020, the dominant landscape types in the Baili Rhododendron National Forest Park were forest and cultivated land, accounting for approximately 95% of the total land use. Forests were not only the dominant landscape type but also the main type in the land use conversion process. The comprehensive dynamic degree of land use in the study area increased from 8.53% to 9.66% during this period, with construction land showing the most significant dynamics and notable changes. (2) Overall, the landscape ecological risk index in the study area decreased over the 20-year period, with high-risk areas showing a trend of shifting from north to south. The distribution of risk was primarily characterized by low-medium, medium, and medium-high risk areas, with slight decreases in the areas of low, low-medium, and high-risk zones, and increases in medium and medium-high risk zones. Among the 1225 ecological risk zones, approximately 96.8% showed a decrease in the risk index, while about 0.03% showed an increase. (3) Spatial autocorrelation analysis revealed that the landscape ecological risk index exhibited positive spatial correlation in each period, although the spatial clustering gradually weakened. High-high (HH) and low-low (LL) zones were concentrated, while low-high (LH) and high-low (HL) zones were scattered. HH and LL zones exhibited high consistency with the distribution range of high-risk and low-risk areas.
